# Comparison of Cochlear Implant Magnets and Their MRI Artifact Size

**DOI:** 10.1155/2020/5086291

**Published:** 2020-01-10

**Authors:** I. Todt, R. Guerkov, H. B. Gehl, H. Sudhoff

**Affiliations:** ^1^Department of Otolaryngology, Head and Neck Surgery, Bielefeld University, Campus Mitte, Bielefeld, Germany; ^2^Department of Radiology, Klinikum Bielefeld, Bielefeld, Germany

## Abstract

**Introduction:**

Recent developments regarding cochlear implant magnets (e.g., a bipolar diametral magnet) and refined surgical techniques (e.g., implant positioning) have had a significant impact on the relation between cochlear implants and MRIs, making the reproducible visibility of cochlea and IAC possible. MRI scanning has changed from a contraindication to a diagnostic tool. Magnet artifact size plays a central role in the visual assessment of the cochlea and IAC.

**Objective:**

The aim of this study is to compare the CI magnet-related maximum artifact sizes of various cochlea implant systems.

**Materials and Methods:**

We performed an in vivo measurement of MRI artifacts at 1.5 and 3 Tesla with three cochlear implant magnet systems (AB 3D, Medel Synchrony, and Oticon ZTI). The implant, including the magnet, was positioned with a head bandage 7.0 cm and 120° from the nasion, external auditory canal. We used a TSE T2w MRI sequence on the axial and coronal plains and compared the artifacts in two volunteers for each tesla strength.

**Results:**

Intraindividual artifact size differences between the three magnets are smaller than interindividual maximum artifact size differences. 3 T MRI scans, in comparison to 1.5 T MRI scans, show a difference between soft artifact areas.

**Conclusion:**

We observed no major difference between maximum implant magnet artifact sizes of the three implant magnet types.

## 1. Introduction

For a long time, MRI observations were a contraindication after a cochlear implantation [1]. With the introduction of headbands, a compromise was found between the need for an MRI scan and the risk of pain and magnet dislocation. Due to the internal magnet, MRI scans can be associated with complications such as pain or dislodged magnets [2–4] at 1.5 T or cannot be performed without the removal of the magnet at 3 T (Series 5 implant, Cochlear Company, Sydney, Australia; Neuro 2, Oticon, Vallauris, France). This observation limits the usability of MRI scans. New implants that contain diametrically bipolar magnets (Synchrony, Medel, Innsbruck, Austria) allow for pain-free MRI scans at 3 T, even without a headband [5].

Recent studies indicate that an MRI-based visual assessment of the internal auditory canal, even after an implantation is performed [6], is possible and that such an evaluation, even for the cochlea, depends on the implant magnet's specific positioning and the MRI sequence used [7]. Todt et al. showed that the internal auditory canal is most visible when a position on the basis of the nasion-external auditory canal-internal magnet angle and the distance between the internal magnet and the external auditory canal are given. An internal magnet position at 90° and 7 and 9 cm, 120° and 9 cm, and 160° degrees and 7 and 9 cm is recommended.

This observation allows for the reproduction of a view on the cochlea's fluid state after the insertion of an electrode. A correlation is shown between electrode position and specific MRI T2 diminishing for perimodiolar electrodes [8]. This correlation even holds true at 3 T for lateral wall electrodes at the basal turn [9].

Clinically, the postoperative MRI follow-up after acoustic neuroma resection and cochlear implantation is an option that depends greatly on the IAC's visibility [10].

Because magnet artifact location inside the patient's head depends on the relation between implant magnet head and scanning unit, it was shown that it can be rotated in various implant positions [7]. The head's position inside the scanner is an additional variable. It was shown that an anteversion (cheek-to-chest) position of the head led to a rotation of the implant magnet artifact away from the cochlea and the IAC [11].

Because two magnet generations are currently available, which offer a pain and headband-free MRI observation [12], it is of special interest if a regular bipolar (first-generation magnet, Oticon ZTI, Vallauris, France) generates a different artifact size than a newly rotatable diametrically bipolar (second-generation magnet, Medel Synchrony, Innsbruck, Austria) or a 3D rotatable cylindrical bipolar (second-generation magnet; Advanced Bionics 3D, Stäfa, Switzerland). Additionally, it is of general interest if a 1.5 T MRI scan generates a different artifact than a 3 T MRI scan.

The current study is an evaluation of CI magnet-related maximum artifact sizes of the three various implant magnets and a comparison of 1.5 T and 3 T MRI scans with comparable T2 TSE sequences.

## 2. Materials and Methods

The observation was performed in the radiology department of Klinikum Bielefeld, Bielefeld, Germany, in 2018 and approved by the local institutional review board (IRB klibi-hno-2/2019).

For the study, the artifacts of an Advanced Bionics 3D Implant (Advanced Bionics, Stäfa, Switzerland), Medel Synchrony Implant (MED-El, Innsbruck, Austria), and a Oticon ZTI (Oticon, Vallauris, France) were compared at 1.5 T for all and 3 T MRI for the first two systems. The implant magnet with the whole implant was placed at a 120° nasion external auditory implant angle and 7 cm from the external auditory canal. The implant was fixed on the volunteer's heads with a tight head bandage. After each scan, the implant's position was controlled and relocated if necessary. The volunteers had a regular anatomy of the head and neck region without any pathology. Three volunteers were scanned 20 times. Volunteers 1 and 2 were imaged with 3 T and volunteers 2 and 3 with 1.5 T.

The imaging was performed with 1.5 T MR imaging (Achieva, Philips, Best, NL) with T2-weighted TSE sequences: TSE T2 2 D: TR: 3000 ms, TE 120 ms, slice thickness 1.5 mm, voxel size 0.449 mm, and F0V 180.19 slices.

The imaging was also performed with 3.0 T MR imaging (Achieva, Philips, Best, NL) with T2-weighted TSE sequences: TSE T2 2 D: TR: 3000 ms, TE 120 ms, slice thickness 0.8 mm, voxel size 0.449 mm, and F0V 230  × 199.35 slices.

In addition, the surveys performed in T1 before each scan were compared for 1.5 T in the coronal and sagittal plains. For the 3 T scans performed in T1, the surveys were performed only in the sagittal plain.

**Figure 1:**
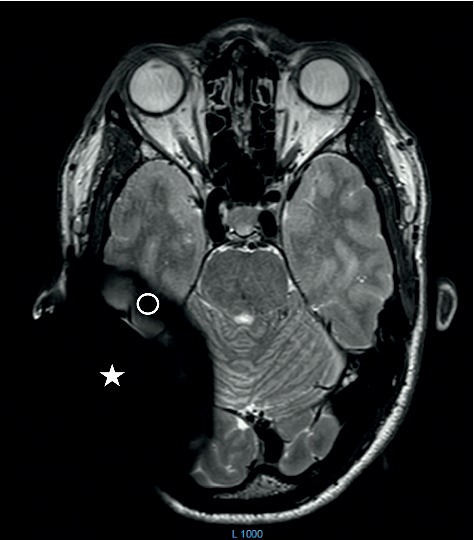
MRI survey pattern of the sagittal plain of a 1.5 T scanning of an Advanced Bionics 3D Implant.

We differentiated between a soft and a hard artifact for the regular 1.5 T and 3 T comparisons. A hard artifact was defined as a completely black area. A soft artifact was defined as the surrounding cloudy inconsistent area (Figure 1). To reach a comparability in terms of point of measurement, the scan with the largest inward directed artifact area was used, which was usually at the height of the upper ocular lens.

From this height, 4 measurements were performed.

**Figure 2:**
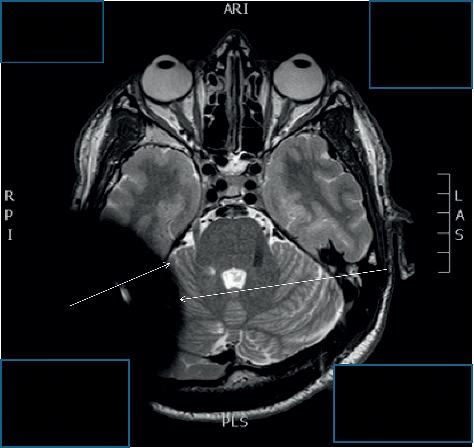
Example for an artifact estimation based on a contralateral measurement and an ipsilateral measurement in the axial plain.

**Figure 3:**
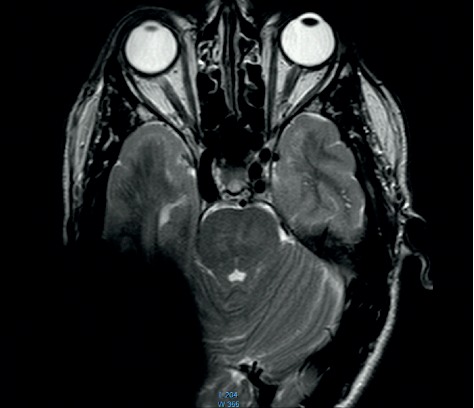
MRI pattern of the axial plain of a 1.5 T scanning Advanced Bionics 3D Implant.

- From the contralateral helix and the underlying cortical bone to the soft artifact (1) and to the hard artifact (2) (Figure 2)
- From a virtual ipsilateral cortical bone to the hard artifact (3) and to the soft artifact (4) (Figure 3)

For the statistical analysis, the absolute differences between distinct volunteers were compared to the absolute differences between any pair of brands using a two-sided, heteroscedastic Student's T-test.

## 3. Results

**Figure 4:**
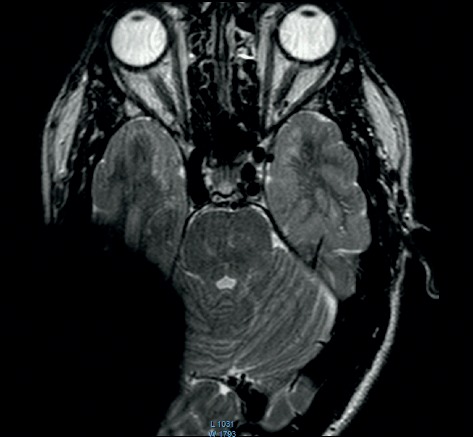
MRI pattern of the axial plain of a 1.5 T scanning Medel Synchrony Implant.

**Figure 5:**
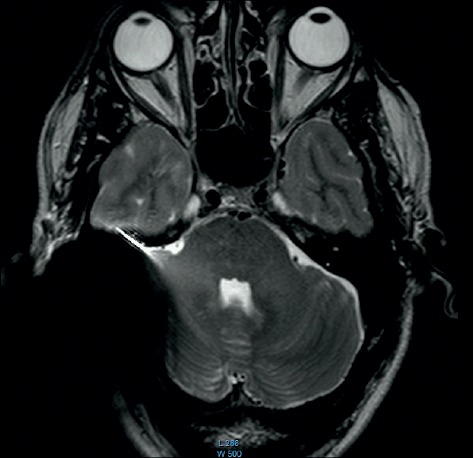
MRI pattern of the axial plain of a 1.5 Tscanning Oticon ZTI Implant.

A comparison of the artifact sizes at 1.5 T for all brands shows a similar artifact size for both volunteers in the ipsilateral measurement. The measurement of the contralateral-side artifact's size differs between the volunteers due to their different head sizes. Figures 3–5 show the exemplary axial 1.5 T T2 TSE scans of the brands in one volunteer.  Table 1 presents the individual measurements.

**Figure 6:**
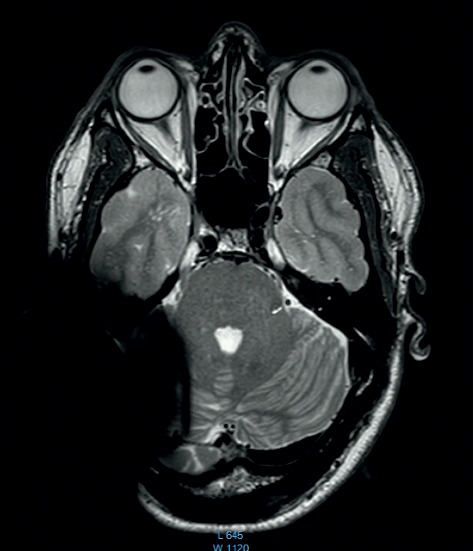
MRI pattern of the axial plain of a 3 Tscanning Advanced Bionics 3D Implant.

**Figure 7:**
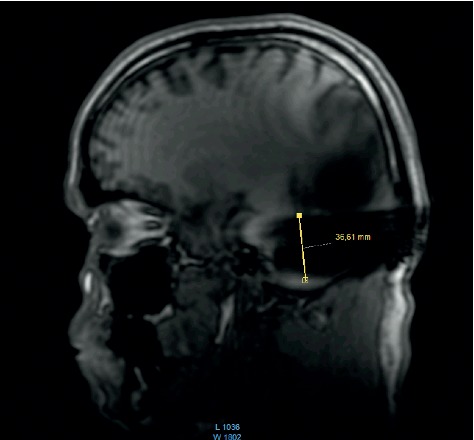
MRI pattern of the axial plain of a 3 T scanning Medel Synchrony Implant. White star indicates the hard artifact area. Circle indicates the soft artifact area.

Comparing the artifact size at 3 T for the two brands shows a similar artifact size for both volunteers in the ipsilateral measurement. Even here, the measurement from the contralateral-side artifact's size differs between the volunteers due to their different head sizes. Figures 6 and 7 show the exemplary axial 3 T T2 TSE scans of the brands in one volunteer.  Table 2 presents the individual measurements.

**Figure 8:**
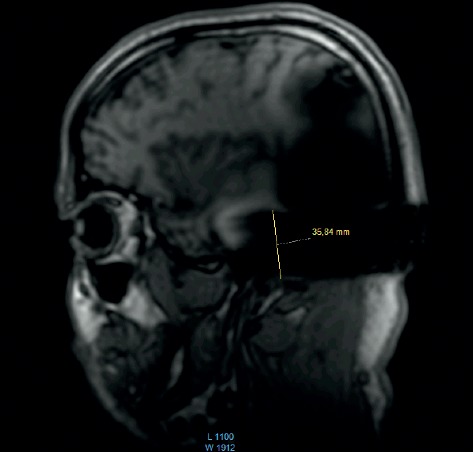
MRI survey pattern of the sagittal plain of a 1.5 T scanning of a Medel Synchrony implant.

**Figure 9:**
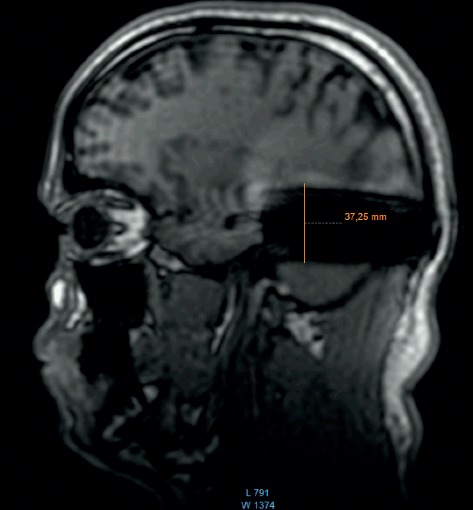
MRI survey pattern of the sagittal plain of a 1.5 T scanning of an Oticon ZTI Implant.

**Figure 10:**
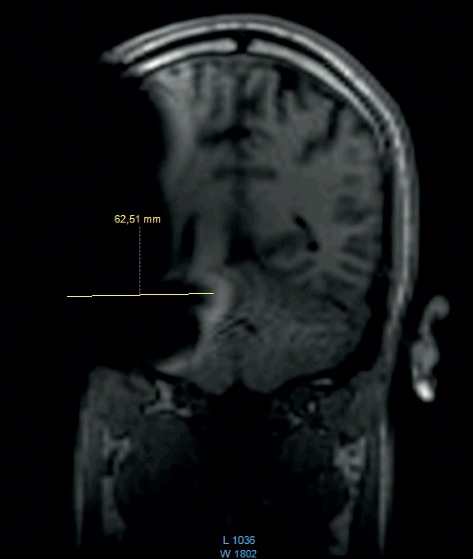
MRI survey pattern of the coronal plain of a 1.5 T scanning of an Advanced Bionics 3D Implant.

**Figure 11:**
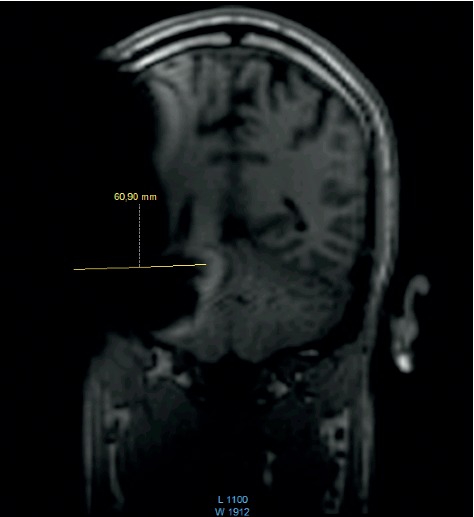
MRI survey pattern of the coronal plain of a 1.5 T scanning of a Medel Synchrony Implant.

**Figure 12:**
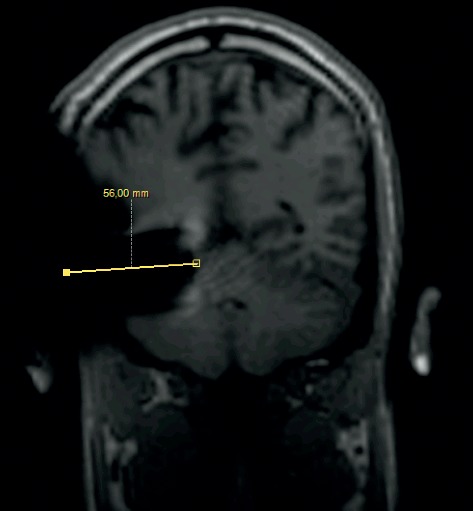
MRI survey pattern of the coronal plain of a 1.5 T scanning of an Oticon ZTI Implant.

The comparison of the 1.5 T surveys shows almost identical artifact sizes for all brands at the sagittal plane (Figures 1, 8, and 9; Table 1). The coronal plane demonstrates even here, with one exception, comparable artifact size for all brands (Figures 10–12; Table 1).

**Figure 13:**
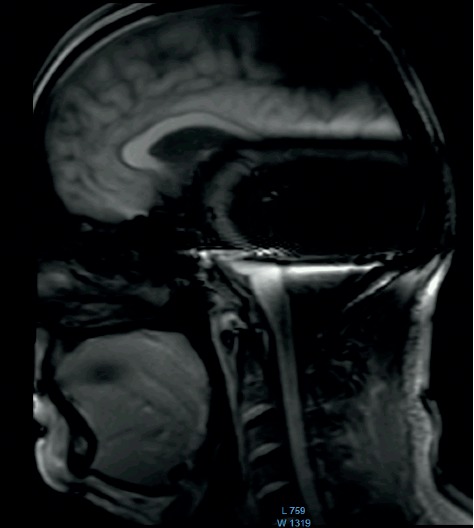
MRI survey pattern of the sagittal plain of a 3 T scanning of an Advanced Bionics 3D Implant.

**Figure 14:**
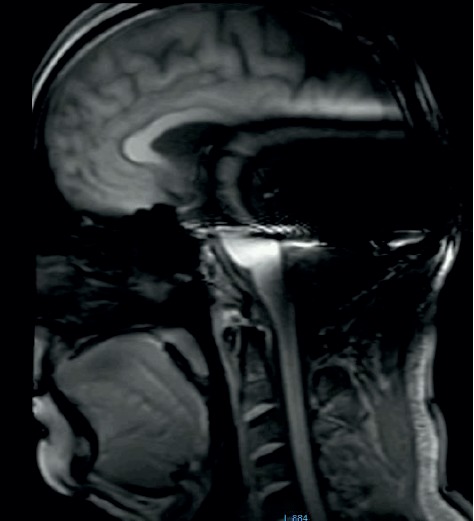
MRI survey pattern of the sagittal plain of a 3 T scanning of a Medel Synchrony Implant.

The results at 3 T sagittal surveys were similar. Even here, we observed no difference between the two brands at 3 T (Figures 13 and 14).

A comparison of the artifacts between 1.5 T and 3 T T2 TSE scans shows a comparable artifact size of the soft and hard artifacts. Looking at the soft artifact area, we observe at 1.5 T only a slight difference in the blackening of the hard artifact (Figures 3–5). The soft artifact at 3 T shows a less intense blackening than the hard-artifact area (Figures 6 and 7).

Next, the artifact size data were analyzed for the differences between different volunteers and for the differences between the brands. For this, the absolute difference between the values of two volunteers was measured under the same condition (1.5 T or 3 T), for the same artifact type (hard, soft, survey sag, and survey corona), and using the same brand (AB, Medel, or Oticon) were calculated revealing 28 values (*n* = 28). These absolute differences were compared to the absolute differences between each pair of brands (AB-Medel, AB-Oticon, and Medel-Oticon) measured for the same volunteer for the same artifact type (hard, soft, survey sag, and survey corona) under the same condition (1.5 T or 3 T), which summed up to 48 values (*n* = 48). This analysis revealed that the differences between distinct volunteers (8.14 mm ± 11.33 mm) were significantly greater than the differences between distinct brands (3.09 mm ± 3.03 mm) (*p*=0.031). The maximum absolute difference between individual volunteers was 4.9 mm, whereas the maximum difference between any pair of brands was 1.2 mm. This indicates that the MRT artifact size is rather influenced by the individual physical disparities of imaged patients than the choice of the device brand.

## 4. Discussion

Cochlear implantation is the treatment of choice in cases of deafness and profound to severe sensorineural hearing loss. Over time, indications changed from bilateral to unilateral and asymmetrical hearing loss in patients with residual hearing. With the increase in cochlear implant patients, the number of MRIs increased.

Recent developments in the relation between MRI, CI systems, and the implant magnet's surgical positioning allow for postoperative MRI scanning and evaluation of the cochlea and IAC without any discomfort.

The development of a diametrically bipolar implant magnet (Medel Synchrony, Innsbruck, Austria) allows for a pain-free MRI scan without a headband at 3 T [5]. The additional positioning of the magnet >8 cm from the external auditory canal allows for the visual assessment of the internal auditory canal and cochlea [7].

This positioning allows for the estimation of the electrode's position [8], even at 3 T [9]. This finding is very important for the audiological rehabilitation of acoustic neuroma patients because it allows for the combination of neuroma surgery, cochlear implantation, and MRI follow-up [10].

The recent introduction of a system with a 3D rotatable cylindrical bipolar implant magnet (AB 3D, Advanced Bionics, Stäfa, Switzerland) can be assumed to have similar properties in terms of a pain-free 3 T MRI scan. Both systems' physical principles are comparable because they are based on the self-direction of the internal magnet inside the MRI scanner's transferred magnet field. This direction prevents torsion of the implant or the magnet against skin and periost. This principle is different from that of the first-generation magnet of the Oticon ZTI (Vallauris, France). Even this system allows for pain-free MRI scans at 1.5 T [11] but not for 3 T due to the risk of demagnetization. The physical principle allowing for a pain-free MRI scan is here transmitted by a screw fixation of the implant. This mechanism prevents torsion of the implant during the MRI scan.

Related to evaluation of vertigo, unclear performance loss, exclusion of an infarction [13], or new occurrence of tumors after the implantation is very important. Even in cases of previous intralabyrinthine schwannoma or acoustic schwannoma, MRI scanning as a tumor follow-up is of central importance [10]. Therefore, the magnet-artifact's behavior is important for the control of the internal diminishing.

Multiple factors can affect the IAC's and cochlea's visibility. Besides the choice of MRI sequence [7] and the implant magnet's position in the head, the head's position inside the MRI scanner is a third factor [11]. A chin-to-chest position (anteversion) allows for the rotation of the artifact away from the IAC and the cochlea.

A possible fourth factor is the implant magnet itself. At 1.5 T and an axial comparison of the artifact soft and hard artifacts, the size ranges from ipsisoft 3.9 to 5 cm and ipsihard 3 to 4.4 cm. Contralateral measurements range from soft 8.5 to 10 cm and contrahard 10.3 to 10.9 cm. Here, the intraindividual difference (maximum 1.2 mm (volunteer 3 ipsihard Oticon vs Medel)) is smaller than the interindividual difference (maximum 1.5 cm (volunteer 3 vs volunteer 2, contra-AB)) (Table 1). Comparison of the artifact sizes shows that differences occur mostly in the low part of the range with minor variations between the brands in terms of lowest artifact sizes. Limitations in terms of these measurements are to some degree a virtual line during the ipsilateral measurement as a baseline, and looking at the contralateral measurement, the head size itself is a contributing factor which biases the measurement.

Even at 3 T, an axial comparison of the soft- and hard-artifact sizes reveals a range from ipsisoft 5.3 to 6 cm and ipsihard 3 to 4.1 cm. Contralateral measurements range from soft 10.6 to 11 cm and contrahard 8.5 to 13.4 cm. Here, the intraindividual difference (maximum 0.4 cm (AB vs. Medel; volunteer 2 soft contra)) is smaller than the interindividual (maximum 4.9 cm (volunteer 1 vs volunteer 2; contrahard AB)) (Table 2). Comparison of the artifact sizes shows that differences are mostly in the lower part of the range with minor variations between the brands in terms of lowest artifact sizes. Limitations in terms of this measurement are to some degree a virtual line during the measurement ipsilateral as a baseline, and looking at the contralateral measurement, the head size itself is a contributing factor that biases the measurement.

The comparison between 1.5 T and 3 T artifact sizes shows larger differences in soft artifacts under both conditions (ipsi and contra). Although the 1.5 T artifact shows a less clear difference between soft and hard artifacts, the comparison with the soft artifact seems more valuable. Parameter of the 1.5 T and 3 T T2 TSE 2D sequences is comparable. The difference in terms of resolution (3 T and 0.8 mm vs. 1.5 T and 1.5 mm) has no impact on the artifact's size. It affects resolution and scanning time.

Previous studies have shown similar artifact size between T2 2D and T1 2D [7]. 3D sequences have been shown to allow further reduction of resolution (>0.8 mm) but are associated with larger artifacts [7].

Comparison of the sagittal survey scans shows no difference between the implant magnets. This result holds true for the 1.5 T and the 3 T scans.

Looking at the coronal plain, which was only performed at 1.5 T, a difference between the systems was not observed, with two exceptions. In a single measurement, Oticon ZTI shows a smaller peak artifact, and the area of the whole artifact gives the impression of being smaller. This finding is assumed to be related to the integration of the magnet inside the implant body.

In general, survey sequences are different from clinical sequences because they help adjust the scanner before the relevant scan. They allow for an easy comparison, but sequences are not artifact optimized.

Interindividual differences in artifact size, as found during our observations, can be explained by the difference in the individual implant's underlying area of bone, fluid, and cerebrum.

Since the implant position for all volunteers is controlled for the same position, the situation in terms of artifact size between the different brands is better comparable than in a vivo implanted study (different implant positions). A cadaveric study is not comparable to the in vivo situation in terms of artifacts since the tissue condition has a high impact on the artifact of an MRI (vital tissue > regular fluid and fat saturation).

The performed study has one general limitation: the positioning of the implant above the skin. Because the scalp's thickness can reach 1 cm, the implanted magnet artifact is farther from the IAC and the cochlea, as in the implant situation.

Based on our findings, a recommendation for a specific brand in terms of maximum magnet artifact size cannot be given.

## 5. Conclusion

We observed no major differences between implant magnet maximum artifact sizes of the three implant magnet types.

## Figures and Tables

**Table 1:** Individual measurements at 1.5 T.

		Distance of measurement	AB		Medel		Oticon	
			Ipsilateral	Contralateral	Ipsilateral	Contralateral	Ipsilateral	Contralateral
	Area of measurement	in cm						
Volunteer 3	Hard artifact		3.8	10.9	3.2	10.4	4.4	10.7
	Soft artifact		4.7	10	3.9	9.8	4.8	10
							
	Survey sagittal		3.7		3.6		3.7	
	Survey coronal		6.1		6		6	
Volunteer 2	Hard artifact		3.2	10.3	3	10.8	0.3	10.3
	Soft artifact		5	8.5	4.7	8.6	4.1	9.3
							
	Survey sagittal		3.6		3.5		3.6	
	Survey coronal		6.3		6.1		5.5	

The table shows each measurement for each brand in terms of estimation (ipsilateral and contralateral) and the differentiation between soft and hard . In addition, the survey calculation is added

**Table 2:** Individual measurement at 3 T.

		Distance of measurement	AB		Medel	
			Ipsilateral	Contralateral	Ipsilateral	Contralateral
	Area of measurement	in cm				
Volunteer 1	Hard artifact		3	8.5	3	8.9
	Soft artifact		5.3	10.9	5.3	11
	Survey sagittal		5.8		5.8	
Volunteer 2	Hard artifact		4.1	13.4	4	13.3
	Soft artifact		6	11	5.8	10.6
	Survey sagittal		5.8		5.7	

The table shows each measurement for each brand in terms of estimation (ipsilateral and contralateral) and the differentiation between soft and hard artifact. In addition, the survey calculation is added.

## Data Availability

The data used to support the findings of this study are available from the corresponding author upon request.
